# LGR4 and Its Role in Intestinal Protection and Energy Metabolism

**DOI:** 10.3389/fendo.2015.00131

**Published:** 2015-08-25

**Authors:** Ziru Li, Weizhen Zhang, Michael W. Mulholland

**Affiliations:** ^1^Department of Surgery, University of Michigan Medical Center, Ann Arbor, MI, USA; ^2^Department of Physiology and Pathophysiology, Peking University Health Science Center, Beijing, China

**Keywords:** LGR4, R-spondin, digestive system, energy metabolism, diabetes, colon cancer

## Abstract

Leucine-rich repeat-containing G protein-coupled receptors were identified by the unique nature of their long leucine-rich repeat extracellular domains. Distinct from classical G protein-coupled receptors which act via G proteins, LGR4 functions mainly through Wnt/β-catenin signaling to regulate cell proliferation, differentiation, and adult stem cell homeostasis. LGR4 is widely expressed in tissues ranging from the reproductive system, urinary system, sensory organs, digestive system, and the central nervous system, indicating LGR4 may have multiple functions in development. Here, we focus on the digestive system by reviewing its effects on crypt cells differentiation and stem cells maintenance, which are important for cell regeneration after injury. Through effects on Wnt/β-catenin signaling and cell proliferation, LGR4 and its endogenous ligands, R-spondins, are involved in colon tumorigenesis. LGR4 also contributes to regulation of energy metabolism, including food intake, energy expenditure, and lipid metabolism, as well as pancreatic β-cell proliferation and insulin secretion. This review summarizes the identification of LGR4, its endogenous ligand, ligand–receptor binding and intracellular signaling. Physiological functions include intestinal development and energy metabolism. The potential effects of LGR4 and its ligand in the treatment of inflammatory bowel disease, chemoradiotherapy-induced gut damage, colorectal cancer, and diabetes are also discussed.

## Introduction

Leucine-rich repeat-containing G protein-coupled receptors (LGRs) are a distinct group of highly conserved proteins of the GPCRs family, characterized by a large extracellular domain (ectodomain) that harbors multiple copies of leucine-rich repeats (LRRs) (1). LRRs represent amphipathic sequences with leucine as the predominant hydrophobic residue and are important for protein–protein interaction (2). The packing of similar repeats allows the formation of a specific hydrogen bond network between neighboring repeats to form a unique tertiary structure (3). LRRs are involved in ligand binding (4), connected via a cysteine-rich region to a seven-transmembrane (TM) domain responsible for heterotrimeric G protein activation (5).

LGRs are divided into three subgroups (groups A–C) (1, 6). Group A receptors include FSH receptor (LGR1), LH receptor (LGR2), and TSH receptor (LGR3), which recognize follicle-stimulating hormone (FSH), luteinizing hormone (LH), and thyroid-stimulating hormone (TSH), respectively (7, 8). These receptors contain seven to nine LRRs in their ectodomains and long hinge regions connecting the LRR domains to TM domains. Group C LGRs have similar number of LRRs but contain a low-density lipoprotein receptor class A domain motif at the N terminus and a short hinge region between the LRR domain and the 7TM domain. Group C LGRs include RXFP receptor 1 (LGR7) and RXFP receptor 2 (LGR8), recognizing relaxin and INSL3 (insulin-like peptide 3), respectively (1, 7, 8). The group B receptors include LGR4, LGR5, and LGR6, which are characterized by a long ectodomain containing 17 LRR repeats (6, 8) flanked by the N-terminal LRRNT region and the C-terminal LRRCT region (8). Group B receptors play crucial roles in embryonic development and are involved in several types of cancer (9). They have also drawn significant attention recently because of their roles in adult stem cells, especially when LGR5 and LGR6 were identified as stem cells markers in multiple adult tissues (10–12).

The ligands of LGR4-6 remained unidentified for a prolonged time. In 2011, the secreted R-spondin proteins (Rspo1-4) were identified as the endogenous ligands for these receptors to regulate cell proliferation, differentiation, and adult stem cell maintenance through the activation of Wnt signaling pathways (13, 14). Details on the binding between R-spondins and LGRs, and the subsequent intracellular signaling pathways are still under investigation. Recent studies have also revealed a relationship between LGR4 and energy metabolism in areas ranging from food intake and obesity to lipid metabolism.

## Identification of LGR4

In 1998, based on the knowledge that large G protein-coupled seven-TM receptors for LH, FSH, and TSH contain LRRs which interact with glycoprotein ligands, and the theory that the putative glycoprotein hormone receptor sequences are conserved in *Drosophila* and sea anemone (15, 16), human sequences related to the sea anemone and *Drosophila* glycoprotein hormone receptors (15, 16) were sought using expression sequence tags. Fragments of two new mammalian receptors in the subfamily of leucine-rich repeat-containing G protein-coupled receptors (LGR) were identified. Adding to the three known LGRs, these two new mammalian receptors were named LGR4 and LGR5 (17).

The full-length cDNAs for these novel receptors were isolated using RT-PCR and repeated screening of sub-libraries from rat ovary or human placenta enriched with each receptor cDNA. LGR4 cDNA from rat ovary consists of 3,504 base pairs with a predicted open reading frame (ORF) of 951 amino acids, whereas LGR5 from human placenta has 4,208 base pairs with a 907 amino acids ORF (17). Similar to three known glycoprotein hormone (LH, FSH, and TSH) receptors, LGR4 and LGR5 are characterized by multiple LRR sequences. The ectodomains of LGR4 and LGR5 are composed of 17 LRR motifs, each 22–24 amino acids in length (17). In contrast to the restricted tissue expression pattern of known gonadotropin and TSH receptors, these new receptors were found in multiple tissues (17).

Identification of this expanding family of LGRs promoted studies to identify putative ligands and to unravel the evolutionary origin of proteins in this subfamily of receptors.

## Ligands of LGR4

The R-spondin (Rspo) protein family is a group of four secreted proteins (Rspo1-4) that were isolated as strong potentiators of Wnt/β-catenin signaling (18–20). These proteins share 40–60% identity between each other and a similar structure with a cysteine-rich furin-like domain preceding a thrombospondin-like domain (21, 22). Despite their similarity, the four known Rspos serve in different developmental events. Rspo1 regulates sexual development; Rspo2 modulates development of limbs, lungs, and hair follicles; Rspo3 is involved in placenta development; and Rspo4 affects nail development (23).

Beginning in 2011, several groups have demonstrated that R-spondins (Rspo 1-4) are endogenous ligands for LGR4 and LGR5. A fusion gene construct (mRspo1-Fc), encoding the mature form of mouse Rspo1 and the Fc fragment of mouse IgG2a, is biologically active (24). When incubating cells expressing LGR4 or LGR5 with mRspo1-Fc at 4°C (to prevent internalization), a strong signal indicative of binding was observed on the cell surface. Whole-cell competition binding assay showed that recombinant Rspo1-4 could compete with mRspo1-Fc for binding to LGR4 and LGR5 (25). The results of binding analyses indicate that Rspo1-4 can bind to LGR4 and LGR5 with Rspo2 having the highest affinity to both receptors. Using β-catenin-responsive reporter assay (26), cells transfected with LGR4 or LGR5 displayed a dramatic increase in the potencies of Rspo1-4, ranging from 10- to 1,000-fold, on Wnt/β-catenin signaling in the presence of exogenous Wnt3a (25).

Experiments using an unbiased screening strategy have also identified LGR4 and LGR5 as receptors of Rspo proteins (27). Depletion of LGR4 completely abolishes Rspo1 signaling, while overexpression of LGR4 potentiates Rspo1-4 signaling. Rspo1 interacts with the extracellular domain of LGR4 and LGR5 (27). Further, Rspo1 does not induce coupling between LGR4 and heterotrimeric G proteins, suggesting that LGR4 transmits Rspo signaling through a novel undefined mechanism independent of G protein signaling. This likely contributes to the relatively long time taken to deorphanize LGR4. This study further supports the conclusion that Rspo potentiates Wnt/β-catenin signaling through LGR4 and LGR5, which is described in detail in Section “Intracellular Signaling of LGR4.”

## Binding of R-Spondins to LGR4/5

**Figure 1:**
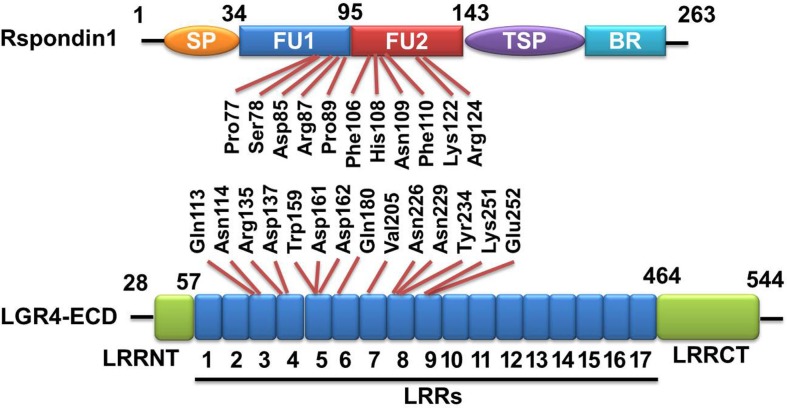
**Rspondin1-LGR4 linear sructure illustration**. Linear structure of Rspondin1 and LGR4-ECD. Rspondin1 consists of a signal peptide (SP), two adjacent cysteine-rich furin-like domains (FU1/2), a common thrombospondin (TSP-1) motif and a basic amino acid-rich (BR) domain. LGR4 ectodomain (LGR4-ECD) are characterized by 17 leucine-rich repeats (LRRs) flanked by the N-terminal LRRNT region and the C-terminal LRRCT region. The main interacting residues of LGR4-ECD are LRR3-9 [refer to Ref. (4, 5) for detailed information].

**Figure 2:**
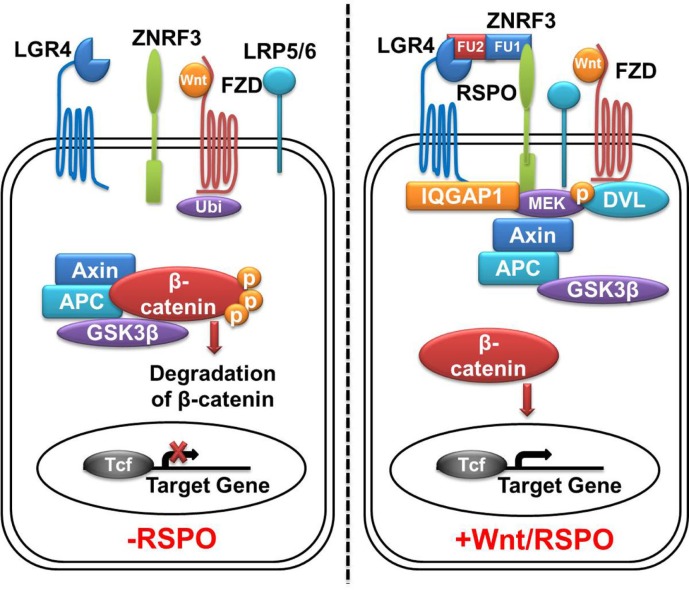
**LGR4-Wnt/β-catenin signaling pathway**. In the absence of Rspos, membrane E3 ubiquitin ligases ZNRF3/RNF43 ubiquitinates the (Frizzled) FZD receptor for degradation, Wnt signaling activity is blunted. Cytoplasmic β-catenin is degradated by the β-catenin destruction complex, leading to no β-catenin complex formation with T-cell transcription factor (Tcf) and subsequent silence in active transcriptional response. In the presence of Rspos, simultaneous binding of LGR4 and ZNRF3 inhibits the ubiquitination of FZD receptor, meanwhile, LGR4 recruits IQGAP1 and increases its affinity to DVL, leading to the formation of supercomplex with Wnt signalosome. This allows β-catenin accumulation in cytoplasm, followed by translocation into the nucleus and activation of TCF target genes. LGR4, leucine-rich repeat-containing G protein-coupled receptors 4; ZNRF3, zinc and ring finger 3; FZD, Frizzled class receptor; Rspos, R-spondins; LRP5/6, low-density lipoprotein receptor-related protein 5**/**6; Ubi, ubiquitination; DVL, disheveled.

The extracelluar domain (ECD) of LGR4 exhibits a twisted horseshoe-like structure. Rspo1 adopts a flat and fold architecture and is bound in the concave surface of LGR4 through electrostatic and hydrophobic interactions (28). All the Rspo1-binding residues are conserved in LGR4-6, suggesting that LGR4-6 bind R-spondins through an identical surface. R-spondin proteins have the same structural organization. They have two adjacent furin-like domains (FU1 and FU2) at the amino terminus, and a thrombospondin (TSP) domain close to the carboxyl terminus (29) (Figure 1). Sequence similarities of furin-like domains and TSP domain of four Rspo proteins from different species are high, for example, the identities of Rspo1 protein sequences between human and mouse is 94% (from Pubmed Blast), suggesting R-spondin proteins have conserved functions. A fragment containing two furin-like domains of R-spondin is sufficient to activate Wnt signaling, and both furin-like domains are required for the signaling activity of R-spondin (18, 30). In addition to LGR4/5, various membrane proteins have been reported to bind to R-spondin, including Wnt receptors Frizzled (20) and LRP6 (20, 31), Kremen (32), Syndecan 4 (33), and membrane E3 ubiquitin ligases ZNRF3/RNF43 (34). For example, Xie et al. reported that mutation of furin-like domain 1 (FU1) (R66A or Q71A) abolished the interaction between Rspo1 and ZNRF3, without affecting the interaction between Rspo1 and LGR4, while mutation of two residues of the furin-like domain 2 (FU2) (F106A or F110A) blocked the interaction between Rspo1 and LGR4, without affecting the interaction between Rspo1 and ZNRF3 (29). These results suggest that Rspo1 binds to ZNRF3 and LGR4 through distinct domains: the FU1 domain is involved in ZNRF3 binding, whereas the FU2 domain is involved in LGR4 binding. In the absence of Rspo, ZNRF3/RNF43 ubiquitinates the FZD receptors for degradation, resulting in low Wnt signaling activity. Rspo1 can bind to both ZNRF3 and LGR4 to induce their dimerization (34). In the R-spondin-LGR4–ZNRF3 complex, LGR4 serves as the engagement receptor to recruit R-spondin to ZNRF3. ZNRF3 serves as the effector receptor. Inhibition of ZNRF3 by R-spondin potentiates Wnt signaling. The R-spondin-LGR4/5–ZNRF3/RNF43 complex represents a fascinating example of a secreted protein regulating receptor turnover by targeting membrane E3 ubiquitin ligases (29) (Figure 2).

## Intracellular Signaling of LGR4

Studies have suggested that interaction between Rspo proteins and LGR4 potentiates canonical Wnt/β-catenin signaling, but does not activate Gi, Gs, or Gq pathways. Structurally, LGR4 and LGR5 are quite similar to other LRR-containing GPCRs which are coupled to heterotrimeric G protein signaling by ligand binding. The intracellular signaling pathway by which Rspo and LGR4 potentiate Wnt/β-catenin signaling remains largely unknown.

By examining proteins co-immunoprecipitated with LGR4, Kendra et al. identified IQGAP1 and IQGAP3 as potential candidates that could mediate the intracellular signaling of Rspo–LGR4 to the Wnt signalosome (35). Interaction between LGR4 and IQGAP1 occurs between the 7TM domain of LGR4 and the rasGAP-related domain (GRD) of IQGAP1. Stimulation of LGR4 by Rspos increases the affinity of IQGAP1 to disheveled (DVL), leading to the formation of a supercomplex between Rspo–LGR4 and the Wnt signalosome (35). Potentiation of Wnt signaling requires the MEK1/2-binding domain of IQGAP1, which provides the hint that LGR4-bound IQGAP1 brings in MEK1/2 to phosphorylate LRP6. In this configuration, IQGAP1 not only engages MEK1/2 to phosphorylate LRP5/6 but significantly enhance canonical Wnt signaling. IQGAP1 also recruits actin-polymerization complexes through binding to neural Wiskott–Aldrich syndrome protein (N-WASP) and mDia1 to coordinate actin dynamics (35), which is critical to the control of cell adhesion and migration (36–38). This dual-mechanism model provides an explanation for the pleiotropic functions of Rspo–LGR4 signaling in normal and cancer development, particularly for the crucial role of LGR4 in tubule elongation and branching in multiple organs (Figure 2).

Other signaling pathways have also been reported for LGR4. LGR4 (GPR48) participates in the development of the male epididymis and efferent ducts through regulation of ERalpha expression via the cAMP/PKA signaling pathway (39). LGR4 (GPR48) knockout suppressed ATF4, a key transcription factor in erythropoiesis, in midgestation fetal livers through the cAMP–PKA–CREB pathway, suggesting that GPR48 regulates erythropoiesis through ATF4 (40).

## LGR4 in Intestinal Development

### Expression in stem cells

A common feature of the LGR4/5/6 receptors is their expression in distinct types of adult stem cells. LGR5 is a marker for resident stem cells in Wnt-dependent compartments, including the small intestine, colon, stomach, and hair follicles (9). LGR6 serves as a marker of multipotent stem cells in the epidermis (12). LGR4 is widely expressed in proliferating cells (41).

The expression of LGR4 was investigated using an animal model with disrupted LGR4 gene by a trap vector carrying two biological markers, β-geo (a fusion between bacterial β-galactosidase and neomycin phosphotransferase) and a placental alkaline phosphatase (PLAP) (42). Due to the perinatal lethality of homozygosity for this insertion, LacZ and PLAP activity patterns in heterozygous mice were investigated. In embryonic day (E) 14 embryos, LGR4 was strongly labeled in eyes, tongue surface, olfactory epithelium and vomeronasal organ, ribs, esophagus, cartilage condensation of vertebrae, umbilical cord, medulla oblongata, pons, and Rathke’s pouch (41). In adults, strong LacZ staining signals were detected in cartilage, kidney, adrenal gland, salivary glands, and testis, while lower intensity was observed in a wide range of organs (41).

### LGR4 in digestive system

At E15 and at birth, LacZ activity was detected in the pseudo-stratified epithelium and intervillus progenitors, respectively. In adults, epithelial expression of LGR4 was found along the crypts, but not in the villi by using X-gal staining and *in situ* hybridization (41). In the crypts, LGR4 expression was found above the Paneth-cell zone, in the transit-amplifying (TA) cell region, in crypt basal columnar cells, and co-localized with some Paneth cells. Outside the epithelium, LGR4 is expressed in the mesenchyme and smooth-muscle layers, intestinal subepithelial myofibroblasts and enteric neurons. A similar expression pattern was found in the duodenum and colon (41).

Another study has validated the presence of LGR4 immunoreactivity in Paneth cells which were distinguished by large secretory granules in the cytoplasm (43). Similar staining was also noted in crypt stem cells which are sandwiched between Paneth cells (44). No significant staining of LGR4 was found beyond the cells in the crypts. The intense LGR4 positive staining signal observed in Paneth cells and stem cells in the crypt region of mouse intestine were consistent with that of LGR4 mRNA distribution determined using lac-Z alleles (45). In the mouse colon, only diffuse, weak LGR4 immunoreactivity in the cytoplasm was found in all cells from the base to the epithelial surface, with slightly stronger staining at the surface. No distinct staining was found in stem cells located at the crypt base in colon (43).

Intense LGR4 immunoreactivity was also found in the pancreatic islets with no staining in acinar cells. Co-staining of anti-LGR4 and anti-insulin antibody on mouse pancreas sections showed that LGR4 was expressed in all β-cells (43). Moreover, among the three LGR receptors, only LGR4 is expressed in the pancreas based on the analysis of EST data and Northern blotting (46). The intense staining of LGR4 in the islets strongly suggests that LGR4 mediates the effects of its endogenous ligands in the pancreas.

### LGR4 in intestinal development

Intestinal crypts contain LGR5^+^ stem cells and their TA daughter cells, as well as terminally differentiated Paneth cells. Cells exiting crypts terminally differentiate into enterocytes, goblet cells, M-cells, Tuft cells, and enteroendocrine cells, and move up the flanks of the villi to undergo apoptosis upon reaching the villus tips (47). Paneth cells escape the crypt–villus flow by migrating to crypt bottoms where they persist for several weeks (48).

Mice homozygous for the gene trap *LacZ* knock-in allele, referred to as “*Lgr4* knockout”, displayed a hypomorphic phenotype with intrauterine growth retardation and perinatal lethality. Heterozygous mice with a *LacZ* gene trap knocked in the *Lgr4* locus (42) have also been used. Although the timing of crypt development was normal in *Lgr4*-knockout mice, reduction in the crypt depth and epithelial cell proliferation were obvious from postnatal day (P) 15 (49). Differentiation of absorptive, enteroendocrine, and goblet-cell lineages was not modified significantly. However, defects in Paneth-cell differentiation were observed at all postnatal stages, with reduction in Paneth-cell number at P21 and decreased expression of the terminal differentiation markers P-lyzozyme and cryptdin 4 (49), suggesting a key role for LGR4 in postnatal epithelial cell proliferation and terminal Paneth-cell differentiation.

*Ex vivo* experiments have demonstrated that LGR4 is required for the maintenance of crypt stem cells. Crypts cultured from P15 wild-type or heterozygous mice differentiated into multi-fingered organoids after 3 days in culture. After generation of hollow spheres containing mainly stem and TA cells (day 0.5–1), structures grown from P15 *Lgr4*-knockout intestine did not increase further in size and became filled with cellular material (days 1.5–2) (49). *Lgr4*-knockout organoids started to disaggregate between days 2 and 3, and died before day 7. The same phenotype was observed in *Lgr4*-knockout progenitors isolated from newborn mice, when fully differentiated Paneth cells are not yet present (49).

## LGR4 in Energy Metabolism

The presence of LGR4 in hypothalamic energy homeostatic areas and its co-localization with key energy homeostatic neurons suggests that it may contribute to the regulation of energy homeostasis. *In situ* hybridization revealed that LGR4 mRNA is highly expressed in the cortex, hippocampus, amygdala, and the hypothalamus (41). In the cortex, LGR4 mRNA is expressed in layers II and III. In the hippocampus, LGR4 is expressed in CA1, CA2, CA3, and the dentate gyrus (DG).The habenular nuclei (Hbs) of the epithalamus also express LGR4. In the amygdala, LGR4 mRNA is expressed with high levels in the medial amygdaloid nucleus, posteroventral nucleus, and basal lateral amygdaloid nucleus (41). In the hypothalamus, LGR4 is expressed in the ventromedial hypothalamus (VMH), the arcuate nucleus (ARC), median eminence (ME), and the ependymocytes lining the third ventricle (50). The ME and ependymocytes have the highest levels of LGR4 expression, followed by VMH and ARC. The expression pattern of LGR4 in the VMH overlaps that of brain-derived neurotrophic factor (BDNF) (51). Double *in situ* hybridization showed that in the ARC, LGR4 is expressed by most neuropeptide Y (NPY) neurons and proopiomelanocortin (POMC) neurons. NPY neurons express higher levels of LGR4 compared with POMC neurons in the ARC. All POMC neurons in the ME express LGR4 at the highest levels. In the VMH, LGR4 is expressed by the majority of BDNF neurons (50).

Rspo1 and Rspo3, ligands of LGR4, are expressed in hypothalamic energy homeostatic areas (50). Their levels were down-regulated by fasting and up-regulated by the satiety factor insulin, indicating that they might be involved in the regulation of energy homeostasis as anorexigenic factors. The inhibition of food intake observed after intracerebroventricular injection of Rspo1 or Rspo3 support this concept (50). Rspo1 is more potent than Rspo3 in inhibiting food intake. Consistent with this observation, Rspo1 binds to LGR4 with an affinity higher than Rspo3 (25).

GPR48 (LGR4) is critical in development, and Gpr48 mutant mice display early neonatal lethality. Wang et al. have established the Gpr48 (LGR4) hypomorphic mutant mice by microinjecting gene trap-mutated Gpr48 ES cells into C57BL/6 blastocysts. The insertion of the trap vector into intron 1 of the Gpr48 gene resulted in approximately 90% knockdown efficiency in the kidney and adrenal gland of adult LGR4 mutant mice. Approximate half of LGR4 mutant newborns died within 28 h after birth, but no further deaths occurred in the following 20 h (52). Studies of the *Lgr4*-knockout mice have revealed a critical role of LGR4 in lipid metabolism. Relative to wild-type mice fed normal chow diet, both male and female *Lgr4* mutant mice exhibited decreased body weight and body fat content, including epididymal white adipose tissue (eWAT) and inguinal WAT (iWAT), whereas brown adipose tissue (BAT) content remained unaltered (53). Consistent with the lean phenotype, *Lgr4* mutant mice showed improved glucose tolerance and reduced fasting total cholesterol levels. When challenged with a high-fat diet (HFD), both male and female *Lgr4* mutant mice showed a resistance to HFD-induced body weight gain, with improved glucose tolerance and insulin sensitivity (53). Higher O_2_ consumption, CO_2_ production, and body temperature indicated that energy expenditure was elevated in *Lgr4* mutant mice. Compared to the white, large, and unilocular adipocytes comprising WAT in wild-type mice, *Lgr4* mutant mice showed reduced WAT mass with beige color, smaller, and multilocular adipocytes containing increased mitochondrion number. These results indicate an adipocyte phenotype transformation in *Lgr4* mutant mice (52). Consistent with this concept, Ucp1 and other thermogenic genes, including *Pgc-1*α, *Cidea*, *cytochrome c*, *Cpt2*, and *Nrf1*, were significantly increased in eWAT of *Lgr4* mutant mice, and were further enhanced under cold stress or isoprenaline treatment. Beige cell markers CD137 and TMEM26 (54) were also increased in eWAT of *Lgr4* mutant mice after isoprenaline treatment. These results demonstrate that *Lgr4* ablation drives the acquisition of functional brown-like adipocytes in the WAT depots, leading to increased energy expenditure (52).

The association of LGR4 with human obesity has been demonstrated by a case–control study of early-onset obesity in which four SNPs, located in the encoding and flanking regions of the *LGR4* locus, were found to be significantly correlated with body mass index. A low-frequency non-synonymous *LGR4* variant (A750T) was identified more than twice as commonly in obese patients when compared with controls (52). This site in LGRs is highly conserved among different species, and constitutively activated point mutations of the corresponding site in LHR, TSHR, and FSHR have been reported (55–57). The A750T variant showed higher stimulating activity of a CRE-luciferase reporter than wild-type LGR4, suggesting a functional variation. All these observations suggest a contribution of LGR4 to human adiposity.

Another study based on the LGR4 mutant mice described before also indicates a correlation between LGR4 and lipid metabolism (58) in a circadian rhythm-related manner. Resting energy expenditure (RER) is higher in the dark phase than in the light phase in wild type (WT) mice (59), suggesting the existence of a circadian rhythm in substrate utilization for energy during the day, more glucose in the dark phase, and more lipid usage in the light phase. In *Lgr4* mutant mice, the RER was higher than that of their WT littermates during the dark phase with no difference during the light phase (57), suggesting that lack of LGR4 altered the circadian rhythm of lipid metabolism. *Lgr4*-knockout mice consumed less lipids but more sugar compared with WT mice (60, 61). *Lgr4* mutant mice exhibited higher plasma triglyceride levels and lost the rhythmic pattern compared with WT mice. *Lgr4* mutant mice also presented a change in plasma non-esterified fatty acid levels, reflected by lower plasma levels during the light phase and higher levels in the dark phase in comparison with WT mice. Interestingly, loss of LGR4 does not affect clock gene expression in the liver. In WT mice, LGR4 expression in liver was higher during the light phase than the dark phase, presenting a peak at ZT4 and a nadir at ZT16, indicating a circadian rhythm. Lgr4 expression levels in *Lgr4* mutant mice were very low and amplitude was dampened (57). Lack of LGR4 causes an arrhythmic plasma lipid phenotype in mice.

## Therapeutics Potential of Rspos-LGR4

### Treatment for inflammatory bowel disease

As defective epithelial restitution is an important risk factor for inflammatory bowel disease (IBD), it is not surprising that dysfunction of genes involved in intestinal development, proliferation, and differentiation will increase susceptibility to IBD. When Lgr4 hypomorphic mice are subjected to the dextran sulfate sodium (DSS)-induced IBD, a more severe colitis developed in *Lgr4* mutant mice than in WT sex-matched littermates. Higher body weight loss, a hallmark of intestinal inflammation, was observed in *Lgr4* mutant mice. This observation was concordant with a more severe anemia (45). Although the small intestine is not the major target of DSS-induced tissue damage (62), the relative length reduction was significantly increased in *Lgr4* mutant mice, indicating critical functions of Lgr4 in the small intestine. Almost all crypts throughout the small intestine were lost in *Lgr4* mutant mice but remained intact in WT littermates. Histological examination showed dramatically increased signs of colitis, which is characterized by the loss of crypts and the infiltration of leukocytes into the colons of *Lgr4* mutant mice (45). Infiltration of neutrophils was significantly higher in the colon of *Lgr4* mutant mice. Additionally, inflammatory cytokines, such as TNFα, IL6, and IL1, were significantly increased in *Lgr4* mutant mice after DSS administration, suggesting a more severe inflammatory response (45). Significant decreases in Ki67-positive proliferating cells were observed in *Lgr4* mutant intestines during tissue regeneration. No significant alteration of apoptosis was observed either in control conditions or in the recovery period, indicating that LGR4 is responsible for epithelial cell proliferation but not apoptosis during DSS-induced tissue regeneration (45).

Human Rspo1 protein effectively increases survival and proliferation of LGR5^+^ intestinal stem cells *in vitro* through activation of Wnt/β-catenin signaling (63). The mitogenic activity of Rspo1 on intestinal stem cells may be useful in the therapy of IBD because of its stimulating effect on crypt cell growth to accelerate mucosal regeneration. In both acute and chronic phases of colitis in mouse models, administration of Rspo1 protein preserves mucosal integrity in both small and large bowel by stimulating crypt epithelial cell mitosis (64).

### Protective effects of R-spondin in chemoradiotherapy-induced gut injury

Stimulation of Wnt/β-catenin signaling with Rspo1 can ameliorate 5-fluorouracil (5-FU) and radiation-induced gut damage, including radiation-induced gastrointestinal syndrome (RIGS) (64–66). Recently, Zhou et al. have found that combination of Slit2 and Rspo1 could potentially protect gut from chemoradiotherapy-induced damage (67). Slit is a secreted protein which functions through the TM protein Roundabout (Robo) receptor as a chemorepellent in axon guidance and neuronal migration, and as an inhibitor in leukocyte chemotaxis (68). The therapeutic dosage of 5-FU (19), a well-characterized chemotherapy agent, markedly shortened villus length, reduced numbers of LGR5^+^ intestinal stem cells, and Ki67^+^ transient amplifying cells in jejunum. Using Lgr5–enhanced green fluorescent protein (eGFP)–internal ribosome entry site (IRES)–CreERT2 (Lgr5–GFP) mice to detect the intestine stem cells (10), the lethal dose of 5-FU has been found to abolish >90% of GFP^high^ intestinal stem cells. However, a 3-day treatment of rSlit2 or rRspo1 alone protected 40% of GFP^high^ stem cells. Combination rRspo1 plus rSlit2 preserved 80% of GFP^high^ intestinal stem cells, indicating that Slit2 acts synergistically with Rspo, leading to prolongation of overall survival after exposure to lethal doses of chemotherapy (67).

Apc^Min/+^ mice with spontaneous intestinal adenomas were treated with DSS to induce inflammation-related intestinal carcinogenesis, a murine model of multifactorial human colorectal cancer (CRC) (69). Administration of DSS-treated Apc^Min/+^ mice with rSlit2 or rRspo1 alone led to a 20–30% survival rate upon the lethal dosage of 5-FU. Combination of rSlit2 and rRspo1 led to a 60% survival rate (67), demonstrating functional cooperation between Slit2 and Rspo1. Concomitant prolongations of the villus length, augmentations of Lgr5^+^ stem cells and Ki67^+^ transient amplifying cells in the jejunum were observed when animals were treated with a combination of rSlit2 plus rRspo1 (67).

### R-spondin fusion protein in the treatment of colorectal cancer

Colorectal cancer is the fourth most prevalent cancer, accounting for over 50,000 deaths per year in the United States. Approximately 15% of CRCs have microsatellite instability arising from defects in the DNA mismatch-repair system, whereas the other 85% of microsatellite-stable CRCs are the result of chromosomal instability (70).

Using RNA-seq data, Seshagiri et al. have identified 36 rearrangements that result in gene fusions (71), including two recurrent ones. The recurrent fusions found in microsatellite-stable samples involve the R-spondin family members, Rspo2 and Rspo3 (70). Both of them were expected to produce functional Rspos protein. The expression of Rspo2 and Rspo3 in colon tumor samples, containing the fusions, was elevated compared with the tumor samples lacking R-spondin fusions (70). Furthermore, all of the Rspo-positive fusion tumors expressed the potential R-spondin receptors LGR4, LGR5, and LGR6. Additionally, alteration of the Rspo2 gene is linked to CRC in a transposon-based genetic screening in mice (72).Consistent with the elevated expression of Rspo genes observed in human tumors, a 20-fold increase in Rspo2 messenger RNA expression in a mouse tumor carrying a transposon insertion near the Rspo2 transcription start site was detected relative to adjacent normal tissue. These observations indicate that the R-spondins may function as drivers in human CRCs (70). Although further studies are required to fully understand the role of R-spondin fusions in CRC development, they represent attractive targets for antibody-based therapy in CRC patients positive for R-spondin fusions. Other therapeutic strategies that target downstream components of the Wnt signaling cascade might be effective against tumors positive for R-spondin fusions.

### R-spondin in the treatment of diabetes

The development of type 2 diabetes mellitus (T2DM) usually requires the presence of insulin resistance, impaired β-cell function, and the loss of β-cells (73). Type 1 diabetes mellitus (T1DM) is characterized by autoimmune-mediated destruction of β-cells. Novel therapeutic approaches might include expanding β-cell mass. As reported by Wong et al., Rspo1 enhances insulin mRNA levels after stimulation for 12 h. Rspo1 can also regulate insulin secretion in mouse islets. Static incubation of islets with Rspo1 for 2 h induced a significant increase in insulin secretion in a glucose-independent manner (74). Treatment with recombinant mouse Rspo1 also increases MIN6-cell proliferation. Rspo1 induced an increase in BrdU incorporation in insulin-positive cells (74). When treated MIN6 cells were exposed to a mixture of cytokines for 18 h, the level of activated, cleaved caspase3 was significantly increased. The increase in cleaved caspase 3 was prevented by pretreatment with Wnt3a, as well as by Rspo1. A similar observation was made in dispersed murine β-cells. Treatment with cytokines for 18 h significantly increased the number of TUNEL-positive β-cells; pretreatment with Rspo1 significantly reduced cytokine-induced apoptosis (74). These observations suggest that Rspo1 may be a potential novel molecule for the treatment of patients with T2DM or T1DM.

## Conclusion

The importance of LGR4 and its ligands-Rspos in the regulation of canonical and non-canonical Wnt signaling pathways has been established in a variety of *in vitro* and *in vivo* studies using animal models and human genetic analysis. Although significant progress has been made in our understanding of how Rspo binds with LGR4 and regulates the Wnt signaling pathway at the molecular level, the following fundamental questions remain unanswered. How does LGR4 interact with FZD (Wnt receptor) after binding with Rspo proteins? Does LGR4 have a specific intracellular signaling pathway or simply function as a potentiator of Wnt signaling? Additionally, discrepancy between gain and loss of LGR4 function exists. While LGR4-knockout mice showed an improvement in glucose metabolism, Rspo1 has been reported to significantly induce β-cell proliferation and insulin secretion. Further studies on the functions and signaling mechanisms of the LGR4 and Rspo proteins will facilitate the development of therapeutic strategy for human diseases, such as IBD, CRC, and diabetes, by targeting Rspo-LGR4 (Table 1).

**Table 1:** **Roles of LGR4 in intestinal functions and energy metabolism, and summary of the therapeutic potentials of Rspos-LGR4 system**.

Roles of LGR4	Physiological functions	Therapeutics potentials
In intestine	1. Postnatal epithelial cell proliferation and terminal Paneth-cell differentiation	1. Inflammatory bowel disease
	2. Maintenance of crypt stem cells	2. Chemoradiotherapy-induced gut injury
		3. Colorectal cancer
In energy metabolism	1. Inhibition of food intake	1. Obesity
	2. Acquisition of functional brown-like adipocytes in the WAT depots in *Lgr4*^−/−^ mice	2. Diabetes mellitus
	3. Association with human obesity	3. Lipid metabolism
	4. Arrhythmic plasma lipid phenotype in *Lgr4*^−/−^ mice	

## Author Contributions

ZL, WZ, and MM wrote, discussed, and edited the manuscript.

## Conflict of Interest Statement

The authors declare that the research was conducted in the absence of any commercial or financial relationships that could be construed as a potential conflict of interest.
